# Ecosystem-Service Tradeoffs Associated with Switching from Annual to Perennial Energy Crops in Riparian Zones of the US Midwest

**DOI:** 10.1371/journal.pone.0080093

**Published:** 2013-11-06

**Authors:** Timothy D. Meehan, Claudio Gratton, Erica Diehl, Natalie D. Hunt, Daniel F. Mooney, Stephen J. Ventura, Bradford L. Barham, Randall D. Jackson

**Affiliations:** 1 Department of Entomology, University of Wisconsin-Madison, Madison, Wisconsin, United States of America; 2 Department of Forest and Wildlife Ecology, University of Wisconsin-Madison, Madison, Wisconsin, United States of America; 3 Department of Agricultural and Applied Economics, University of Wisconsin-Madison, Madison, Wisconsin, United States of America; 4 Department of Soil Science, University of Wisconsin-Madison, Madison, Wisconsin, United States of America; 5 Department of Agronomy, University of Wisconsin-Madison, Madison, Wisconsin, United States of America; 6 Great Lakes Bioenergy Research Center, University of Wisconsin-Madison, Madison, Wisconsin, United States of America; 7 Nelson Institute for Environmental Studies, University of Wisconsin-Madison, Madison, Wisconsin, United States of America,; University of Missouri, United States of America

## Abstract

Integration of energy crops into agricultural landscapes could promote sustainability if they are placed in ways that foster multiple ecosystem services and mitigate ecosystem disservices from existing crops. We conducted a modeling study to investigate how replacing annual energy crops with perennial energy crops along Wisconsin waterways could affect a variety of provisioning and regulating ecosystem services. We found that a switch from continuous corn production to perennial-grass production decreased annual income provisioning by 75%, although it increased annual energy provisioning by 33%, decreased annual phosphorous loading to surface water by 29%, increased below-ground carbon sequestration by 30%, decreased annual nitrous oxide emissions by 84%, increased an index of pollinator abundance by an average of 11%, and increased an index of biocontrol potential by an average of 6%. We expressed the tradeoffs between income provisioning and other ecosystem services as benefit-cost ratios. Benefit-cost ratios averaged 12.06 GJ of additional net energy, 0.84 kg of avoided phosphorus pollution, 18.97 Mg of sequestered carbon, and 1.99 kg of avoided nitrous oxide emissions for every $1,000 reduction in income. These ratios varied spatially, from 2- to 70-fold depending on the ecosystem service. Benefit-cost ratios for different ecosystem services were generally correlated within watersheds, suggesting the presence of hotspots – watersheds where increases in multiple ecosystem services would come at lower-than-average opportunity costs. When assessing the monetary value of ecosystem services relative to existing conservation programs and environmental markets, the overall value of enhanced services associated with adoption of perennial energy crops was far lower than the opportunity cost. However, when we monitized services using estimates for the social costs of pollution, the value of enhanced services far exceeded the opportunity cost. This disparity between recoverable costs and social value represents a fundamental challenge to expansion of perennial energy crops and sustainable agricultural landscapes.

## Introduction

Agricultural landscapes provide humans with a variety of valuable ecosystem services [1–3]. They provision us with food, fiber, and animal feed. They regulate the quality of our water, sequester greenhouse gases, host beneficial insects and other wildlife, and provide us with a variety of recreational opportunities. Despite the importance of multiple services, agricultural landscapes tend to be designed to maximize only provisioning services such as crop production, as these generate goods that can be sold in existing markets, yielding income for producers and landowners. This tendency has led to dominance by annual crops and a marked decline in other ecosystem services that are often poorly quantified and undervalued [4,5]. For agricultural landscapes to be sustainable, they need to balance provisioning services, which primarily accrue to individuals, with regulating, cultural, and supporting services, which benefit communities more broadly [6,7].

With growing concern about energy independence and atmospheric change, countries are increasingly turning to agricultural landscapes to provision bioenergy feedstocks in order to produce heat, power, and transportation fuels [8,9]. In North America, first-generation bioenergy crops are predominantly high-input, annual monocultures, such as corn (*Zea mays*). Corn produces considerable biomass and economic returns [10]. However, expanded corn production is also exacerbating declines in water quality [11], soil carbon [12], and habitat availability for beneficial insects [13] and other wildlife [14]. Another option for procuring bioenergy feedstocks from agricultural landscapes is to plant second-generation, perennial energy crops, which include a variety of native and non-native grasses and woody plants. These cropping systems currently bring lower above-ground yields and incomes [10]. However, recent research suggests that perennial energy crops could provide bioenergy while also enhancing water quality [15], greenhouse gas sequestration [16], and habitat quality for beneficial insects and other wildlife [17–19].

Thoughtful integration of perennial energy crops into existing landscapes will require a quantitative understanding of ecosystem-service tradeoffs. Many ecosystem services derive from spatially-dependent processes, so quantifying tradeoffs will require spatially-explicit land-use scenarios and ecosystem service models [5,20,21,22]. To date, there have been few spatially-explicit analyses of tradeoffs among bioenergy crops, and most existing studies have focused on a limited set of services [11,18,19,23]. Here, we report results from a modeling study that explored how strategic replacement of annual with perennial-grass energy crops could affect a diverse set of ecosystem services. We used a suite of models to estimate the effects of spatially-explicit scenarios on the (1) annual net income of feedstock producers generated on focal land, (2) annual net energy yield from focal land, (3) annual phosphorous loading to local surface water, (4) carbon sequestered below ground over 20 years, (5) annual nitrous oxide emissions from focal land, (6) relative abundance of pollinating insects on non-focal cropland, and (7) potential for crop-pest suppression by beneficial arthropods on non-focal cropland. Our primary objectives in this study were to demonstrate an approach for quantifying tradeoffs between multiple ecosystem services as perennial energy crops are added to agricultural landscapes, and to explore how the magnitude of these tradeoffs changes across a study region where physical, ecological, and economic conditions vary considerably.

## Methods

### Study system

**Figure 1:**
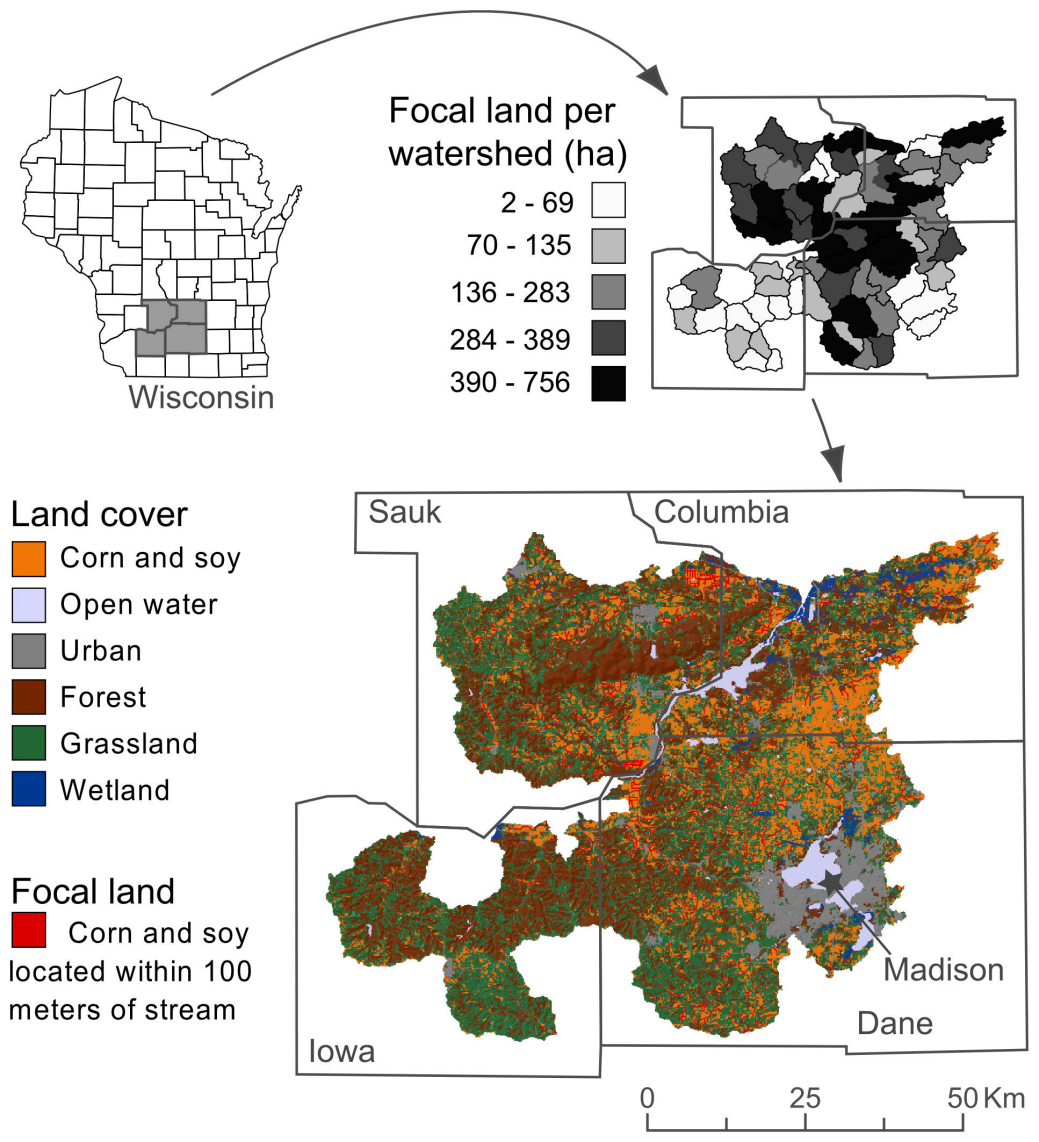
Study system. Land-use patterns, focal-land location, and focal-land area in 67 study watersheds in southern Wisconsin, USA.

Our modeling study was focused on Columbia, Dane, Iowa, and Sauk counties in southern Wisconsin, USA (Figure 1). This region is dominated by agricultural activities, though landscape characteristics vary considerably along a southeast to northwest gradient. The southeastern portion of the region is relatively flat, with deep, fertile soils of glacial origin. Here, landscapes are characterized by large fields of annual monocultures. The northern and western portion of the region is an older unglaciated landscape, more diverse in terms of topography and soil quality. Relatively small fields of annual monocultures occur on better soils along ridge tops and river bottoms, pasture and forage crops occupy poorer soils on moderate slopes, and the steepest slopes are usually covered in woodland.

Within the four-county region, we narrowed our study to 67 watersheds (12-digit USGS Hydrologic Unit Code). We used a derivative of the USDA Cropland Data Layer [24] to determine land use in these watersheds. This derived GIS (geographic information system) map had 100-m resolution and identified land in continuous corn production (70,820 ha), corn-soybean (*Glycine max*) rotations (22,607 ha), corn-alfalfa (*Medicago sativa*) rotations (6,983 ha), other agricultural crops (6,786 ha, mostly alfalfa), urban development (33,316 ha), forests (144,108 ha), perennial grasslands (169,585 ha, mostly pasture, hayfields, and conservation grasslands), and wetlands (9,396 ha, Figure 1). Rotations were determined by comparing the Cropland Data Layer pixel values for the years 2007-2010.

Like other parts of the Midwest, the regional focus on provisioning of food and animal feed has led to declines in surface-water quality from soil erosion and nutrient runoff [25,26]. The region is home to Madison, a city of approximately 220,000 people, located on the shores of several large lakes, within a metropolitan area of nearly 570,000 people. Given the proximity of people and lakes, surface-water quality is an important issue, and several efforts are underway to reduce both point-source and non-point-source pollution [27]. Local concern over water quality was a major factor behind our selection of land for two distinct bioenergy scenarios.

### Land-use scenarios

Of the roughly 100,000 ha under continuous corn and corn rotations, we applied a “continuous-corn scenario” and “perennial-grass scenario” to the same 16,727 ha of “focal land” (Figure 1). Focal land was identified with a GIS as land under continuous corn or corn rotations that was also within a 100-m buffer of streams included in the Hydrography Geodatabase of the Wisconsin Department of Natural Resources [28].

In the continuous-corn scenario, we converted all 16,727 ha of focal land to continuous corn production. This scenario resembles business as usual because, as described above, a majority of the focal land is already under continuous corn management. In the continuous-corn scenario, we assumed that all of the grain and 38% of the stover [10] was harvested for ethanol production. Dedicating all grain to energy production, while simplistic, is not unreasonable given that the focal land represents roughly 17% of the corn and corn-rotation land in the study area and that, nationally, approximately 40% of the annual corn crop goes to ethanol production [29]. A stover harvesting rate of 38% was based on the specifications of current harvesting equipment [10], balanced with the importance of returning some stover to the soil to maintain soil fertility [30].

The perennial-grass scenario represented a departure from business as usual. In this scenario, we converted all 16,727 ha of focal land, currently in continuous corn or corn rotations, to production of a generic perennial-grass energy crop. This scenario was meant to represent a replacement of first-generation, annual energy crops with second-generation, perennial energy crops. A wholesale replacement of first-generation energy crops with second-generation varieties is unlikely in the near term given current federal mandates for grain-ethanol production [31]. However, it is a plausible long-term scenario if future energy policies encourage a transition from grain-based to cellulosic fuels. With these two contrasting land-use scenarios established, we used a variety of methods to estimate economic and environmental outcomes.

### Annual biomass production

Many of the outcomes estimated in this analysis were dependent upon the annual yields of energy crops on focal land. Crop yield estimates (Mg ha^-1^, dry mass) were derived from “representative yields” reported in the USDA SSURGO soil geodatabase [32]. SSURGO representative yields are based on the reported yields of crop producers and the experiences of county conservationists and extension agents, and assume aggressive management practices. These yield estimates are meant to represent site averages, and reflect inherent differences in soil productivity across the region. Actual annual yields are expected to vary with weather patterns and management intensity, and to increase with technological advances.

Annual above-ground biomass production for the continuous-corn scenario was estimated from SSURGO representative corn yields. Yields (bu acre^-1^, 15.5% water by mass) were converted to above-ground plant production (Mg ha^-1^, dry mass) assuming 0.0215 Mg dry corn bu^-1^ and a 1:1 grain-to-residue ratio [33]. We increased SSURGO yield estimates by 20% to account for yield increases due to technological advances that have occurred since SSURGO yields were estimated. Annual above-ground biomass production for a generic, perennial-grass energy crop (Mg ha^-1^, dry mass) was estimated from SSURGO representative yields for grass-legume hay (t ac^-1^, dry mass), specifically an orchard grass (*Dactylis glomerata*) and clover (*Trifolium pratense*) mix.

One limitation of using SSURGO representative yield is that it is not estimated for all map units in the SSURGO geodatabase. When representative yield was not given for a map unit, we estimated a value from a multiple regression of representative yield versus representative slope (%), soil depth (mm), silt content (% within top 30 cm), and cation exchange capacity (cec, mEq 100 g^-1^ soil within the top 30 cm) from map units where all five estimates were present. Under these circumstances, annual above-ground biomass production of corn (Mg ha^-1^, dry mass) was estimated using (N = 1158 map units): Corn production = 3.08 - 0.11(slope) + 0.02(soil depth) + 0.10(silt) + 0.04(cec). Standard errors for the coefficients were 0.214, 0.007, 0.001, 0.002, and 0.003, respectively; the whole-model R^2^ was 0.76 (Figure S1). Annual above-ground biomass production for perennial grass (Mg ha^-1^, dry mass) was estimated using (N = 1096 map units): Perennial production = 2.20 - 0.07(slope) + 0.02(soil depth) + 0.07(silt) + 0.03(cec). Standard errors for the coefficients were 0.179, 0.005, 0.001, 0.002, 0.003, respectively; the whole-model R^2^ was 0.76 (Figure S1). Corn and perennial-grass yield estimates were converted to a raster layer with 100-m resolution for further processing.

### Annual net income

Annual net income generated on focal land ($US ha^-1^) was estimated in a two-step process. The first step was to calculate a “simple net income” from grain production as gross revenues minus production costs. Gross revenues were calculated using annual yield estimates (Mg ha^-1^), along with recent market prices for corn grain ($300 Mg^-1^ dry grain) and low-quality hay ($100 Mg^-1^ dry stover and perennial grass). Production costs ($1,124 ha^-1^ for continuous corn with stover removal, and $412 ha^-1^ for perennial grass) were based on crop enterprise budgets [10]. If all of the farmland in our study area had been in cash-grain farms, then this simple net income estimate would have sufficed for the economic services associated with focal land. However, much of the farmland in the study area is associated with integrated livestock operations.

Integrated livestock operations, especially dairy farms, derive additional value from corn land. This value is related to reduced transportation costs, improved nutrient and feed management, and other economies of scope associated with livestock activities [34]. These factors increase the economic returns from corn land, not only for dairy farmers but also for neighboring farms that are associated with these operations through rental or contractual arrangements. To account for the increased value of corn land to integrated livestock operations, we multiplied our simple net income estimates by an “adjustment factor”, described below, of 1.32, reflecting a 32% increase in the value of corn land due to connections with the dairy sector.

We developed the adjustment factor using data (2010 through 2012) from the Agriculture Financial Advisor Database from the Center for Dairy Profitability at the University of Wisconsin-Madison [35] and the Commodity Cost and Return Database for Wisconsin from the USDA Economic Research Service [36]. The adjustment factor was also developed in two steps. The first step involved calculating a ratio of net returns from corn land on dairy farms, relative to that on cash-grain farms. The second step involved calculating the adjustment factor as the net-return ratio multiplied by the proportion of corn land in our study area that is linked to dairy enterprises.

To obtain an upper bound for the net-return ratio, net returns from corn land on dairy farms were calculated as the on-farm net income per cow ($676 cow^-1^) multiplied by the number of cows per unit of farm area (2.16 cows ha^-1^), giving a value of $1,457 ha^-1^ [35]. The USDA’s estimate for the average annual net return from corn-grain production for the same four-county study area is $606 ha^-1^, giving a net-return ratio of $1,457 ÷ $606 = 2.40. This represents an upper bound because it credits the corn land with all of the added profitability from dairy farming, without attributing any of the gain to other factors of production such as pasture or hay land, management, or other inputs. For this study we assumed a net-return ratio of 1.70, the midpoint between a lower bound of 1.00 and an upper bound of 2.40.

According to the 2007 Census of Agriculture [37], dairy farms account directly for approximately 30% of the corn land in the study area. This is a lower-bound estimate for the amount of corn land associated with dairy production. An upper-bound estimate comes from previous studies suggesting that 60% of grain and forage land in Wisconsin is linked to dairy operations [38]. Again, we split the difference between 30 and 60%, using 45% as a reasonable mid-point estimate for the percent of corn land associated with dairy production in our study area. 

The final adjustment factor was determined by adjusting the net-return ratio, 1.70, for the proportion of corn land associated with dairy farms, 0.45, giving an adjustment factor of 1.32. Given that the final adjustment factor was derived from two midpoint estimates, we assessed how our general conclusions would change if the value was off by up to ± 50%.

### Annual net energy yield

The net energy embodied in the ethanol and coproducts (e.g., dry distiller's grains for animal feed) produced annually with biomass from each pixel of focal land (MJ ha^-1^) was calculated as the energy value of ethanol and coproducts minus the lifecycle energy requirements for production. Energy output and input estimates for this analysis came directly from a published lifecycle assessment [39].

Energy embedded in ethanol was calculated as the yield (Mg ha^-1^) times a mass-to-fuel conversion factor (400 L Mg^-1^ dry corn grain, 380 L Mg^-1^ dry stover and grass) times the energy content of ethanol (21.20 MJ L^-1^) [39]. The energy embedded in coproducts was assumed to equal 20% of the energy embedded in grain ethanol, and 16% of the energy embedded in cellulosic ethanol [39]. The lifecycle energy requirements to produce ethanol and coproducts were divided into agricultural and biorefinery stages. For the agricultural stage, we used an average value of 18.92 GJ ha^-1^ for corn-grain production and 7.41 GJ ha^-1^ for stover harvest and grass production [39]. To distribute the energetic costs of the biorefinery stage across the focal land, we assumed an energy input of 15.24 MJ L^-1^ grain ethanol and 1.71 MJ L^-1^ cellulosic ethanol (the difference reflects energy produced at the biorefinery using leftover biomass from cellulosic ethanol production [39]) and multiplied these values by yield and the appropriate mass-to-fuel conversion.

### Phosphorus pollution

Annual phosphorus loading to the watersheds in our study area (kg P per watershed) was estimated using the InVEST Nutrient Retention model [21,40]. Briefly, this model estimates the amount of nutrients leaving a pixel of land via runoff, the direction of runoff flow, the uptake of runoff nutrients in neighboring pixels, and the ultimate deposition of nutrients into streams. Inputs to this model included (1) a digital elevation model, (2) GIS raster layers for average annual precipitation, soil depth, soil plant-available water content, average annual potential evapotranspiration, and land cover, and (3) a table that provides estimates of nutrient export coefficients and nutrient filtering capacity for each land-cover type.

The digital elevation model (30-m resolution) was from the USGS National Elevation Dataset. Mean annual precipitation data (mm yr^-1^) was from an 800-m resolution raster layer of precipitation normals [41]. Soil depth (mm) and soil plant-available water content (unitless) were estimated for each soil map unit using the SSURGO geodatabase [32]. Mean annual potential evapotranspiration (mm yr^-1^) came from a 16-km resolution raster layer from the United Nations Food and Agriculture Organization [42]. Land-cover data was the 100-m resolution crop rotation layer described above. Inputs were converted to GIS raster layers when necessary and all were resampled to 100-m resolution. We used the same set of model parameters and phosphorous export coefficients (Table S1), slightly adjusted from suggested defaults [43], for all scenario evaluations. Adjustments were made to reconcile modeled phosphorous loadings with empirical measures from the literature [44]. When phosphorous-loading estimates from our implementation of the InVEST model were compared with those estimated by the PRESTO model of the Wisconsin Department of Natural Resources [45], we attained a correlation coefficient of 0.79 (Figure S2).

### Below-ground carbon sequestration

In this study, below-ground carbon on focal land was the sum of soil organic carbon in the top 30 cm (SOC) and carbon in below-ground live plant biomass. Sequestration of below-ground carbon on focal land was estimated over a 20-year period from changes in SOC and changes in carbon in below-ground live plant biomass.

Changes in SOC over 20 years were estimated using the approach of West et al. [46]. This approach starts with a baseline SOC estimate and uses empirical rates of SOC change that are dependent upon both projected land use and baseline SOC estimates. Baseline SOC (Mg ha^-1^) was derived from representative organic matter (%) and bulk density (g cm^-3^, measured at 1/3 bar) estimates from the SSURGO geodatabase [32]. Percent organic matter was multiplied by 0.0058 to obtain proportion SOC. Proportion SOC was then multiplied by bulk density, 30 cm depth, 100,000,000 cm^2^ ha^-1^, and 0.000001 Mg g^-1^ to obtain Mg SOC ha^-1^.

Once baseline SOC was estimated, we determined land-use-specific carbon accumulation factors. For continuous corn, a weighted-average carbon accumulation factor of 1.11 was used, based on change factors of 1.00 for conventional-till, 1.10 for reduced-till, and 1.21 for no-till management [46], along with adoption rate proportions of 0.39 for conventional-till, 0.23 for reduced-till, and 0.38 for no-till management [47]. Note that empirical carbon accumulation factors for corn were based on measurements taken under various rates of stover removal. Thus, carbon accumulation estimates under the continuous-corn scenario, where 38% of stover is regularly removed, may be biased high. For perennial grasslands, an accumulation factor of 1.63 was assumed, based on previous studies demonstrating SOC gain when land is converted from cultivation to perennial pasture [46].

Carbon accumulation factors were then multiplied by adjustment factors ranging from 0.20 to 1.20. These adjustment factors were calculated from a function in West et al. [46] that uses baseline SOC content. When calculated adjustment factors fell below 0.2 or above 1.2, they were set to 0.2 or 1.2, respectively, to prevent extrapolation. Finally, adjusted carbon accumulation factors were multiplied by baseline SOC to obtain Mg SOC ha^-1^ in 20 years.

Carbon in below-ground live biomass in 20 years was calculated using above-ground biomass production estimates (Mg ha^-1^, dry mass, described above) and root-to-shoot ratios of 0.18 for corn [48] and 3.43 for perennial grasses [49]. The carbon fraction of below-ground live biomass was assumed to be 0.50 [50].

### Annual nitrous oxide emissions

Annual nitrous oxide emissions from focal land were estimated using the empirical model of Bouwman et al. [51]. This equation estimates annual N_2_O-N emissions from information on land-cover type, annual fertilization rate, soil texture, soil carbon content, soil drainage characteristics, and soil pH. Annual fertilization rates were set to 168 kg N ha^-1^ for continuous corn and 56 kg N ha^-1^ for perennial grasses. Soil characteristics were estimated for each pixel of focal land using the SSURGO geodatabase [32]. SSURGO soil texture was classified as coarse, medium, or fine, based on definitions in Bouwman et al. [52]. Soil carbon content was calculated as percent soil organic matter times 0.58. Soil drainage classes included poor drainage and good drainage, based on definitions in Bouwman et al. [52]. Before processing, soil characteristics layers were rasterized to 100-m resolution for overlay with land-cover and fertilization data. The output of the model was multiplied by 1.57 to convert kg N_2_O-N to kg N_2_O.

### Pollinator abundance

An index representing the relative abundance of pollinators was computed for non-focal cropland in the study region using the InVEST Crop Pollination model [40,53]. Briefly, this model estimates the ability of a landscape to support pollinator foraging and reproduction based on landscape composition and the ability of focal pollinators to move between habitat patches. Inputs to this model include (1) a table of focal bee species that includes their seasonal activity patterns (early, middle, and late summer), nesting habits (soil, wood or stem, cavity, and hive), and travel distances (meters from nest) and (2) a table of habitat-quality scores that reflect the ability of land-cover types to support pollinator nesting and foraging during different parts of the season [40,53].

The focal species used in this analysis included 50 species of bees commonly found in agricultural habitats in southern Wisconsin (Table S2). Activity patterns and nesting habits for focal species was from Wolf and Ascher [54]. Travel distances were from an allometric equation [55] based on species intertegular span, a reproducible measure of body size. Habitat-quality scores were average values estimated by a team of five local insect ecologists (Table S3). The InVEST model output that we employed was an index of relative pollinator abundance that ranges from 0 (few pollinators) to 1 (many pollinators). This index has not been calibrated with pollinator abundance in our region, although it has been shown to be proportional to flower visitation in other systems [53].

### Pest suppression

The potential for crop-pest suppression by predatory arthropods was estimated for non-focal cropland in the study region using an empirical model from Meehan et al. [19]. This model was applied in a moving-window analysis using information on the land cover of each focal pixel and the proportion of the surrounding landscape in perennial grassland. The output of the model (biocontrol index, BCI) is an index that ranges from 0 (low biocontrol potential) to 1 (high biocontrol potential). The index is computed as: BCI = 0.25 + 0.19(crop type) + 0.62(proportion grassland in landscape), where crop type equals 0 for continuous corn and 1 for perennial grass, and the landscape extends 1,500 m from a focal pixel. The index is not directly calibrated with crop loss, although it is negatively related to insecticide use in the US Midwest [19].

## Results and Discussion

### All focal land combined

**Figure 2:**
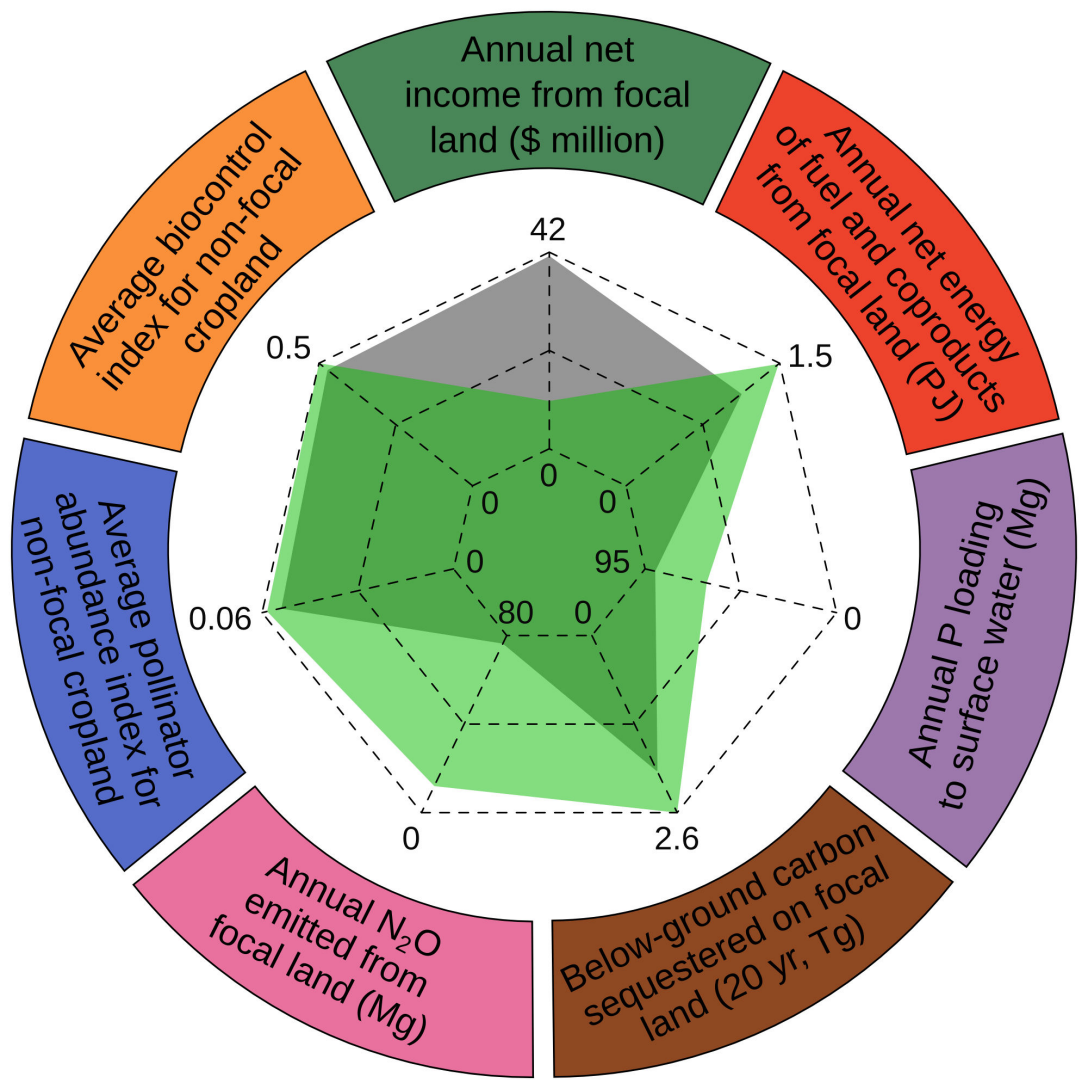
Ecosystem services from focal land. Seven ecosystem services derived from 16,727 hectares of focal land under continuous-corn (gray polygon at center) and perennial-grass (green polygon at center) bioenergy scenarios. Note that axes for phosphorus pollution and nitrous oxide emission are reversed so that the most positive environmental outcomes are consistently furthest from the origin.

The primary objective of this study was to explore ecosystem-service tradeoffs that accompany targeted replacement of annual energy crops with perennial-grass energy crops. The first service that we examined was provisioning of producer income. Previous research suggests that replacement of annual with perennial energy crops will result in a considerable drop in producer income [10]. Similarly, we found that the net income generated in the perennial-grass scenario ($10.2 million) was 75% lower than that generated in the continuous-corn scenario ($40.9 million) (Figure 2). The difference of $30.7 million highlights the large opportunity cost that comes with replacing annual with perennial energy crops. When $30.7 million is divided by the area of focal land (16,727 ha), the mass of grass biomass produced (170,938 Mg), or the number of people living in the study region (approximately 575,326 individuals), this opportunity cost translates to $1,835 ha^-1^, $179 per Mg^-1^, and $53 per person, respectively. From a different perspective, $30.7 million is about 10% of the total net income of farms in the study area (approximately $297 million in 2007 [56]).

The second service we examined was provisioning of energy in the form of ethanol and coproducts. Previous research on the energy balance of biofuel systems suggests that the energy return on investment is higher for cellulosic than for corn-grain ethanol production [39]. Similarly, we found that total annual net energy produced from focal land under the perennial-grass scenario (1.49 PJ yr^-1^) was 33% higher than that of the continuous-corn scenario (1.12 PJ yr^-1^) (Figure 2). The difference (371 TJ yr^-1^) occurred despite corn’s ability to produce greater amounts of above-ground biomass per hectare, and reflects the relatively low energy input and relatively high energy recycling potential of perennial energy crops [39].

We also modeled the effects of the scenarios on several regulating services and disservices. Previous empirical [57] and modeling [58,59] work has shown that soil and nutrient retention is improved when annual crops are replaced with perennial crops, especially along waterways. Similarly, we found that the regulation of water quality, a key consideration in focal-land selection, was greatly enhanced under the perennial-grass scenario (Figure 2). Specifically, annual phosphorus loading to surface water in the region was 25.90 Mg lower in the perennial-grass scenario (64.4 Mg P yr^-1^) than in the continuous-corn scenario (90.3 Mg P yr^-1^). According to Lathrop et al. [25], an equivalent 29% reduction in phosphorus loading in the region's largest lake would reduce the probability of severe blue-green algae blooms (>5 mg L^-1^) by nearly 50%, from 0.42 to 0.22.

Previous empirical [12] and modeling [23] work demonstrates that perennial cropping systems emit considerably less greenhouse gas than annual cropping systems. Using two different metrics, we found that the potential for climate regulation was considerably improved under the perennial-grass scenario. For example, our estimate of below-ground carbon stocks on focal land in 20 years was 2.51 Tg C for the perennial-grass scenario, compared to 1.93 Tg C for the continuous-corn scenario (Figure 2). The 583-Gg difference translates to a 30% increase in carbon sequestration, and a 2.14-Tg reduction in atmospheric carbon dioxide over 20 years. Assuming a linear increase in below-ground carbon stocks over this period [46], this translates to 107 Gg of additional CO_2_ sequestered per year. We also estimated that the change in crop type and reduced nitrogen input of the perennial-grass scenario would bring an 84% reduction in annual nitrous oxide emissions from focal land (Figure 2). Given the global warming potential of nitrous oxide, the 61.3-Mg difference between the perennial-grass scenario (11.7 Mg N_2_O yr^-1^) and the continuous-corn scenario (73.1 Mg N_2_O yr^-1^) represents an annual emissions reduction equal to 19.0 Gg CO_2_.

Previous work has shown that plant and animal taxa are relatively more abundant and diverse in agricultural areas with relatively high perennial habitat cover [60,61]. Similarly, the ability of the landscape to support beneficial insect activity increased under the perennial-grass scenario (Figure 2). For example, the average pollinator abundance index for non-focal cropland adjacent to focal land was 11% higher in the perennial-grass scenario than in the continuous-corn scenario. Likewise, the average biocontrol index for non-focal cropland was 6% higher for the perennial-grass scenario than the continuous-corn scenario. These increases demonstrate how energy-crop choices can have impacts beyond their immediate location, affecting ecosystem services provided by mobile organisms that traverse agricultural landscapes. 

In sum, we found that a single ecosystem service, net income provisioning, was negatively affected by strategic placement of perennial-grass energy crops on corn land adjacent to surface water. The remaining provisioning and regulating services were enhanced by this hypothetical land-use change. A general tradeoff between provisioning and other ecosystem services has been reported in several studies [5,22,58,59,62,63]. This tradeoff can be represented as a ratio of the positive change in any given ecosystem service to the negative change in earned income (hereafter, “benefit-cost ratio”). For example, across the region the perennial-grass scenario produced 12.06 GJ of additional net energy, avoided 0.84 kg of phosphorus pollution, sequestered 18.97 Mg of carbon, and avoided 1.99 kg of nitrous oxide emissions for every $1,000 reduction in income. Simple benefit-cost ratios like these describe the central tendency of ecosystem-service tradeoffs. However, they mask considerable variation that becomes apparent when ratios are explored in a spatial context. Understanding spatial variation in benefit-cost ratios could be useful for locating the most economical locations for different energy crops.

### Focal land per watershed

**Figure 3:**
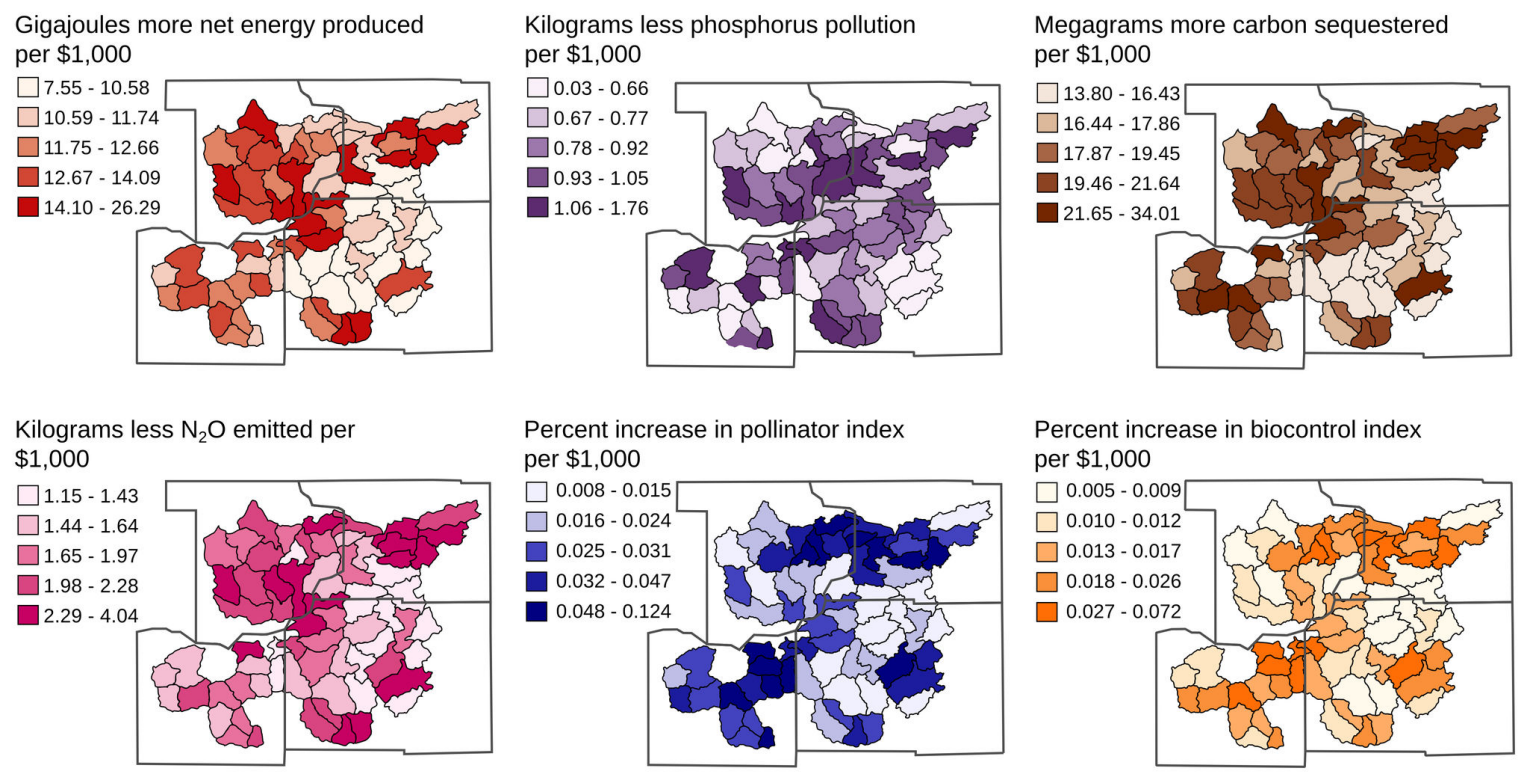
Benefit-cost ratios per ecosystem service and watershed. Benefit-cost ratios (increase in each ecosystem service per $1,000 reduction in producer income) associated with switching from annual to perennial-grass energy crops on focal land in 67 study watersheds in southern Wisconsin, USA.

A second objective of this study was to evaluate how ecosystem-service tradeoffs varied across the study region. When we mapped benefit-cost ratios for each ecosystem service in each watershed, we found considerable variation. The ratio for net energy production varied more than 3-fold, from 7.55 to 26.29 GJ gained per $1,000 reduction in income (Figure 3). The ratio for water quality regulation varied 70-fold, from 0.03 to 1.76 kg P pollution avoided per $1,000 (Figure 3). The ratio for carbon sequestration varied from 13.80 to 34.01 Mg C sequestered per $1,000, while the ratio for nitrous oxide reduction varied from 1.15 to 4.04 kg N_2_O pollution avoided per $1,000 (Figure 3). Finally, ratios for both beneficial insect indices varied more than an order of magnitude across the region (Figure 3). 

In the previous section, we reported regional benefit-cost ratios, calculated irrespective of watershed location. Through analysis at finer spatial scales, we find that ratios can be increased to 26.29 GJ of additional net energy, 1.76 kg of avoided phosphorus pollution, 34.01 Mg of sequestered carbon, and 4.04 kg of avoided nitrous oxide emissions for every $1,000 reduction in income, improvements between 79 and 118%. We explored this spatial variation further to identify hot spots – watersheds, or groups of watersheds, where benefit-cost ratios were relatively high across multiple ecosystem services [56].

**Figure 4:**
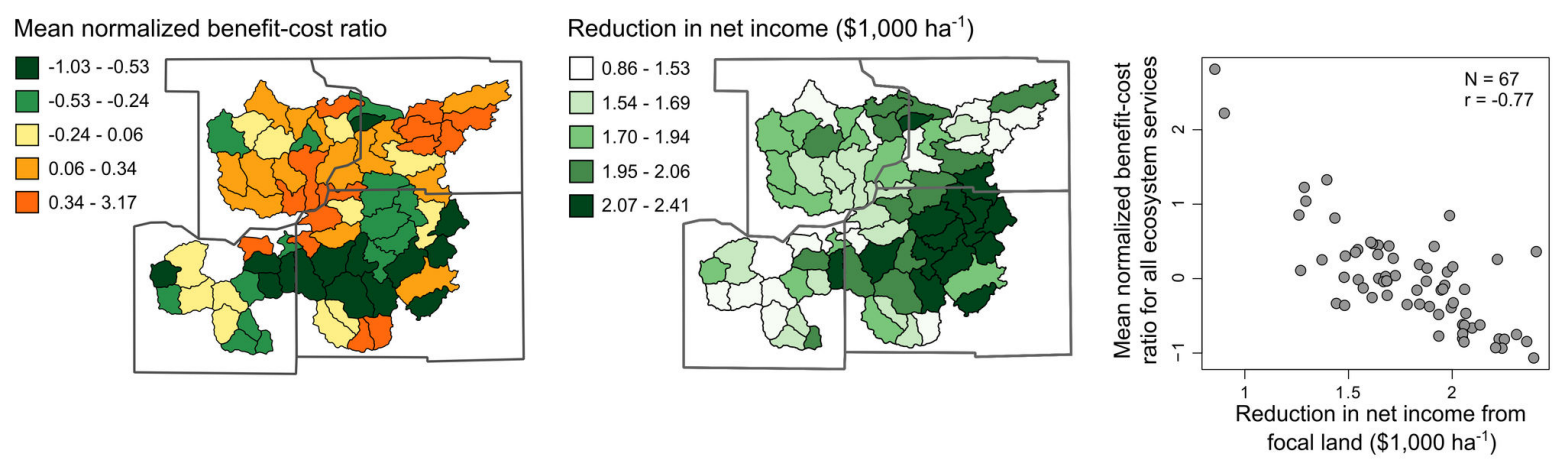
Relationship between generalized benefit-cost ratios and opportunity costs. Spatial (maps) and bivariate (scatterplot, r = Pearson's correlation coefficient) relationships between average normalized benefit-cost ratios and opportunity costs of the perennial-grass scenario (difference in net income between continuous-corn and perennial-grass scenarios) for 67 study watersheds in southern Wisconsin, USA. Average normalized ratios illustrate hotspots – watersheds where switching from continuous corn to perennial-grass brought relatively high environmental benefits for relatively low opportunity cost.

Toward this end, we normalized benefit-cost ratios for each ecosystem service across the 67 watersheds (ratio for each watershed minus the mean ratio for all watersheds, divided by the standard deviation). An investigation of correlations between normal scores showed that benefit-cost ratios were highly correlated for net energy, carbon sequestration, and nitrous oxide emissions (0.70 ≤ r ≤ 0.85, Figure S3). Benefit-cost ratios for pollinator abundance and biocontrol indices were moderately correlated with energy, carbon, and nitrous oxide ratios (0.30 ≤ r ≤ 0.41), and strongly correlated with one another (r = 0.96). The ratio for phosphorus reduction was correlated with that for net energy (r = 0.26), but not with those of other ecosystem services. Using these relationships, we were able to identify watersheds where benefit-cost ratios were consistently high, i.e., tradeoffs between the opportunity cost of perennial-grass energy crops and other ecosystem services were consistently low (Figure 4). By using a simple average to highlight watersheds, we implicitly assumed that all ecosystem services are of equal importance. It would also be possible to compute weighted-average ratios, where weights reflect the relative importance of individual ecosystem services to local stakeholders.

The correlations between benefit-cost ratios across ecosystem services could be due to multiple factors. Perhaps the simplest explanation is that watersheds where ratios were relatively high were the same watersheds where income reductions were relatively low (r = -0.77, Figure 4). Income reductions were relatively low in these watersheds because yield differences between corn and grass are expected to be smaller on lower-quality soils (note the smaller regression coefficients in the perennial production equation). The relatively high performance of grasses on lower-quality soils led to relatively high net energy production on lower-quality soils. At the same time, lower-quality soils are expected to accumulate carbon at a faster rate than higher-quality soils [46]. Similar spatial patterns in beneficial insect indices were likely due to the characteristics of agricultural landscapes in different parts of the study area. For example, abundant fertile soils in the southeastern portion of the study area has led to relatively large expanses of cropland. While switching to perennial grasses along waterways in the southeast does indeed increase beneficial insect indices on adjacent non-focal cropland, the average affect appears small. This is because the ratio of focal to non-focal cropland is considerably lower in the southeast, so the average improvement on non-focal land relative to opportunity costs from focal land becomes relatively small.

### Monetizing ecosystem services

Our analysis used relatively high crop prices ($300 Mg^-1^ dry corn grain and $100 Mg^-1^ dry stover and grass) to evaluate income provisioning under different land-use scenarios. We used these values because they represented crop prices at the time our analysis was conducted. Price forecasts from the USDA suggest that these high prices are temporary [64]. If we assume lower crop prices, then the tradeoffs between income provisioning and other ecosystem services are tempered considerably. For example, if we assume $230 Mg^-1^ dry corn grain, the approximate price forecasted for 2021 by the USDA [64], and $72 Mg^-1^ dry cellulosic biomass, the amount that energy producers might be willing to pay [10], then the total reduction in net income from the perennial-grass scenario would drop from $30.7 million to $21.1 million.

Estimating monetary values for other ecosystem services allows us to evaluate tradeoffs using a common currency. For example, we estimated that the perennial-grass scenario would provision 371 TJ more energy each year than the continuous-corn scenario. If we express additional net energy in terms of liters of ethanol using the conversion factor of 21.20 MJ L^-1^ [39], and assume an ethanol market price of $0.65 L^-1^, then the additional net energy from the perennial-grass scenario can be valued at $11.4 million.

Enhanced water-quality regulation provided by the perennial-grass scenario also has considerable monetary value. For example, water quality programs in the study region are expecting to pay $63.93 kg^-1^ phosphorus for land-use change intended to reduce non-point-source phosphorus pollution [65]. Using this figure, the reduction of 25.9 Mg phosphorus from the perennial-grass scenario can be valued at $1.66 million. Further, a recent economic analysis by the Wisconsin Department of Natural Resources concluded that reduced phosphorus pollution is worth an additional $51.94 kg^-1^ to Wisconsin residents, solely through the effect on real estate values, recreational opportunities, and lake cleanup costs [65]. This translates to an additional $1.35 million value for avoided phosphorus loading in the perennial-grass scenario. Finally, it is possible to assign monetary value to water quality regulation using estimates of people’s willingness to pay for water quality improvement. For example, Johnson et al. [59] used an empirical value of $131 per household as an intermediate estimate of people’s willingness to pay for a 40% reduction in phosphorus loading to surface water in the Midwest. We used their nonlinear method to adjust their willingness to pay value to $111 per household for the 29% reduction in phosphorus loading estimated in our study. Given that there are approximately 240,000 households in the study region, this method gives a value of $26.6 million for the enhanced water quality regulation of the perennial-grass scenario.

It is also possible to monetize the value of climate regulation services. For example, the perennial-grass scenario was associated with an annual greenhouse-gas emissions reduction equivalent to 107 + 19 = 126 Gg CO_2_. The monetary value of these avoided emissions can be assessed using a carbon market price or an estimated social cost of carbon. The current carbon price on the European Climate Exchange is approximately $6 Mg^-1^ CO_2_. A recent estimate for the social cost of carbon (for 2015, given a 3% discount rate) is approximately $38 Mg^-1^ CO_2_ [66]. Multiplying these estimates by the total emissions reduction from the perennial-grass scenario gives values of $756 thousand and $4.79 million per year, respectively.

We can sum monetary values for ecosystem services in two ways to assess tradeoffs associated with the perennial-grass scenario. First, we can calculate a sum using the estimated cost of non-point-source phosphorus pollution management ($1.66 million) and the market value of avoided greenhouse-gas pollution ($756 thousand). This sum, $2.42 million, represents forgone income that could possibly be recovered by producers if they were able to take advantage of current water-quality programs and environmental markets. A second sum can be calculated using the value of additional energy produced ($11.4 million), the willingness to pay for phosphorus pollution avoidance ($26.6 million), and the social cost of carbon ($4.79 million). This sum, $42.8 million, may better represent the value of these services to the larger community.

The disparity between these two estimates for the value of ecosystem services indicates a fundamental challenge for society. The high value of $42.8 million far surpasses the opportunity costs. This is true whether we use low ($21.1 million) or high ($30.7 million) crop prices, and whether we combine high crop prices with low ($27.2 million) or high ($37.5 million) adjustment factors. This suggests that the social value of enhanced ecosystem services from the perennial-grass scenario outweighs the decrease in crop-related income. In contrast, the low ecosystem service value of $2.42 million, which represents what producers might be compensated through participation in current conservation programs and environmental markets, is far below opportunity costs. This is true whether opportunity costs are based on low or high crop prices, and whether we combine low crop prices with low ($13.1 million) or high ($19.5 million) adjustment factors. This disconnect, between the social value of ecosystem services and the amount of money that producers might be compensated to produce them, appears to be a major impediment to the adoption of perennial energy crops in particular, and sustainable agricultural landscapes in general.

### Methodological challenges

Conclusions from this study are based on two hypothetical land-use scenarios and several environmental models. Each environmental model produced first-order estimates based on multiple higher-order estimates. Each of these higher-order estimates had its own inherent variation and statistical uncertainty. In some cases this variation and uncertainty was quantified, and in other cases it was not.

For example, many of the outcomes modeled in this study were derived from yield predictions that were estimated using a database of recollections from agriculturists, with no measures of spatial and temporal variability or statistical uncertainty. When this information was not available, yields were estimated using a multiple regression with confidence intervals that ranged ±11% and prediction intervals that ranged ±62%. We estimated net income from input costs and crop prices assumed to be static over space and time, which clearly is not ideal. Net energy was calculated using 37 estimates of energy inputs and outputs from a lifecycle assessment model, which, itself, was derived from a meta-analysis of other lifecycle analyses. Phosphorus loading was estimated using a water quality model that employed nutrient export coefficients with considerable unspecified variation. Nutrient loading estimates from the water quality model compared favorably to a working model used by state resource managers, but were not a perfect match (r = 0.79, Figure S2). Carbon sequestration was estimated using initial soil carbon values that had unknown spatial variation and measurement error, soil carbon change factors that have standard deviations ranging from 6 to 20%, and estimates (with unreported error) of how soil carbon change is modified by initial soil carbon content. The 95% prediction interval for nitrous oxide emissions was -51 to +107%. The pollinator abundance index was derived from a process-based model that was parameterized with a regression equation for bee travel distances with an R^2^ value of 0.72, and somewhat subjective estimates of habitat quality provided by a group of local insect ecologists. Finally, the biocontrol index was estimated using a statistical model where predictions were bounded by a 95% confidence interval of approximately ±40%.

In addition to estimate uncertainty, our analysis incorporates inaccuracies related to the use of remotely-sensed land-cover data. These inaccuracies are due to two factors. First, the spatial resolution of our data limited our ability to identify, modify, and model the impacts of different land-cover types. Finer grained data (e.g., 56 m resolution) was available, and was more likely to detect landscape features such as narrow hedgerows and stream buffers. However, using finer grained data comes with tradeoffs, making it considerably more challenging to run computationally intensive models, such as water quality models, over large areas. Second, regardless of resolution, land-cover categories derived from remotely-sensed data come with significant classification error [67].

Thus, it should be clear that there is considerable uncertainty in all of the estimates for individual ecosystem services, and that uncertainty is compounded as multiple ecosystem service estimates are compared with one another. Large uncertainty is a common problem in quantitative studies of multiple ecosystem services [5]. However, the same models, parameter values, and land-use maps were used to evaluate both scenarios. So, while absolute values from our models come with considerable uncertainty, qualitative differences between scenarios are likely to be more robust. 

## General Conclusions

Our analysis suggested that replacement of annual with perennial energy crops along Midwestern waterways will enhance a wide range of ecosystem services that are important to society, but will have substantial negative impacts on income provisioning to producers and landowners. A general tradeoff between provisioning and other ecosystem services has been reported many times [5,22,58,59,62,63]. Here, we showed that this tradeoff can vary considerably across space (see also 68) and demonstrate a process for identifying places where environmental benefits of perennial energy crops will come with lower opportunity cost. In their current state, conservation programs and environmental markets likely will not offer enough to compensate producers for lost income. However, estimates for the social value of enhanced ecosystem services were considerably higher than the opportunity costs of switching to perennial energy crops. The difference between social value and opportunity costs would have been even greater if we had included additional ecosystem services in our analysis, such as cultural services, which are often quite valuable, although difficult to monetize [63,69]. Our results underscore the need to incorporate the social value of critical ecosystem services into government policy and market transactions related to bioenergy production.

## Supporting Information

Figure S1 **Fit of empirical crop production models.** Relationships between representative yields for corn and grass-legume hay from the SSURGO database and yields predicted from information on slope, soil depth, silt content, and cation exchange capacity. Pearson's correlation coefficient is denoted by r; N is the number of soil polygons with representative yield estimates.
(TIFF)

Figure S2 **Comparison of phosphorus load models.** Relationship (r = Pearson's correlation coefficient) between InVEST Nutrient Retention and PRESTO model estimates for annual phosphorous loading for 67 study watersheds.
(TIFF)

Figure S3 **Relationships between normalized benefit-cost ratios for multiple ecosystem services.** Normalized ratios were calculated per ecosystem service as the ratio for each watershed minus the mean across watersheds, divided by the standard deviation across watersheds. Spearman's correlation coefficients are given on panels where correlations are statistically significant (P < 0.05).
(TIFF)

Table S1 **InVEST Nutrient Retention model inputs.**
(DOC)

Table S2 **Bee species used in the InVEST Crop Pollination model, along with information on nesting habits and active seasons, and travel distances between nests and foraging areas.**
(DOC)

Table S3 **Average habitat quality scores (0 is worst and 1 is best) for nesting and foraging bees used in the InVEST Crop Pollination model.**
(DOC)
