# Supplementary material for: Ecosystem-Service Tradeoffs Associated with Switching from Annual to Perennial Energy Crops in Riparian Zones of the US Midwest
Source: PLoS One. 2013 Nov 6;8(11):e80093. doi: 10.1371/journal.pone.0080093 (PMC3819318; doi:10.1371/journal.pone.0080093)
Supplement: Table S1 — InVEST Nutrient Retention model inputs. (DOC) [file pone.0080093.s004.doc]

| **Table S1. InVEST Nutrient Retention model inputs.** | | | | |
| --- | --- | --- | --- | --- |
| Land use type | Evapotranspiration (mm) | Root depth (mm) | Phosphorous export coefficient (g/ha) | Phosphorous filtering coefficient (%) |
| Continuous corn | 600 | 2,000 | 2,000 | 15 |
| Corn-soy rotation | 600 | 2,000 | 3,000 | 15 |
| Corn-alfalfa rotation | 600 | 2,000 | 1,500 | 25 |
| Continuous soybeans | 600 | 2,000 | 4,000 | 15 |
| Other annual crops | 600 | 2,000 | 3,000 | 15 |
| Small grains | 600 | 2,000 | 1,500 | 15 |
| Continuous alfalfa | 800 | 3,000 | 100 | 35 |
| Orchards | 700 | 5,000 | 1,500 | 35 |
| Open water | 1 | 1 | 1 | 1 |
| Suburbs | 400 | 1,000 | 1,200 | 15 |
| City | 200 | 500 | 1,200 | 5 |
| Barren | 1 | 1 | 1 | 1 |
| Deciduous forest | 1,000 | 7,000 | 10 | 80 |
| Conifer forest | 1,000 | 7,000 | 10 | 80 |
| Grassland | 700 | 3,000 | 100 | 40 |
| Wetland | 1,000 | 3,000 | 100 | 80 |
