# Supplementary material for: Ecosystem-Service Tradeoffs Associated with Switching from Annual to Perennial Energy Crops in Riparian Zones of the US Midwest
Source: PLoS One. 2013 Nov 6;8(11):e80093. doi: 10.1371/journal.pone.0080093 (PMC3819318; doi:10.1371/journal.pone.0080093)
Supplement: Table S2 — Bee species used in the InVEST Crop Pollination model, along with information on nesting habits and active seasons, and travel distances between nests and foraging areas. (DOC) [file pone.0080093.s005.doc]

| **Table S2. Bee species used in the InVEST Crop Pollination model, along with nesting habits and active seasons, and travel distances between nests and foraging areas.** | | | |
| --- | --- | --- | --- |
| Species | Nesting habit | Active season | Travel distance (m) |
| *Agapostemon splendens* | Soil | Sp, Su | 423 |
| *Agapostemon virescens* | Soil | All | 1,182 |
| *Andrena chromotricha* | Soil | Su, Fa | 535 |
| *Andrena commoda* | Soil | Sp, Su | 1,226 |
| *Andrena peckhami* | Soil | Fa | 803 |
| *Andrena wilkella* | Soil | All | 639 |
| *Apis mellifera* | Hive | All | 2,233 |
| *Augochlorella aurata* | Soil | All | 289 |
| *Bombus bimaculatus* | Hive | All | 4,423 |
| *Bombus borealis* | Hive | All | 4,440 |
| *Bombus griseocollis* | Hive | All | 9,988 |
| *Bombus impatiens* | Hive | All | 5,141 |
| *Bombus rufocinctus* | Hive | All | 7,799 |
| *Bombus sandersoni* | Hive | All | 5,725 |
| *Bombus vagans* | Hive | All | 3,796 |
| *Ceratina dupla* | Wood | All | 255 |
| *Dufourea monardae* | Soil | Su | 255 |
| *Halictus confusus* | Soil | All | 360 |
| *Halictus ligatus* | Soil | All | 600 |
| *Halictus parallelus* | Soil | Sp, Su | 1,556 |
| *Halictus rubicundus* | Soil | All | 412 |
| *Hylaeus modestus* | Cavity | All | 237 |
| *Lasioglossum albipenne* | Soil | All | 346 |
| *Lasioglossum anomalum* | Soil | All | 397 |
| *Lasioglossum bruneri* | Soil | All | 397 |
| *Lasioglossum cinctipes* | Soil | All | 397 |
| *Lasioglossum coriaceum* | Soil | All | 625 |
| *Lasioglossum cressonii* | Wood | All | 286 |
| *Lasioglossum ellisiae* | Soil | Su | 397 |
| *Lasioglossum leucozonium* | Soil | All | 733 |
| *Lasioglossum paradmirandum* | Soil | All | 397 |
| *Lasioglossum pectorale* | Soil | All | 397 |
| *Lasioglossum perpunctatum* | Soil | All | 397 |
| *Lasioglossum pictum* | Soil | All | 275 |
| *Lasioglossum pilosum* | Soil | All | 397 |
| *Lasioglossum pruinosum* | Soil | All | 322 |
| *Lasioglossum quebecense* | Soil | All | 397 |
| *Lasioglossum swenki* | Soil | Su | 397 |
| *Lasioglossum versatum* | Soil | All | 397 |
| *Lasioglossum zephyrum* | Soil | All | 226 |
| *Lasioglossum zonulum* | Soil | All | 943 |
| *Megachile brevis* | Cavity | All | 2,467 |
| *Megachile latimanus* | Cavity | All | 3,651 |
| *Megachile montivaga* | Cavity | All | 1,927 |
| *Megachile pugnata* | Cavity | Su, Fa | 2,001 |
| *Melissodes agilis* | Soil | All | 1,704 |
| *Melissodes bimaculata* | Soil | All | 2,378 |
| *Melissodes desponsa* | Soil | Su, Fa | 2,244 |
| *Melissodes subillata* | Soil | Su, Fa | 1,166 |
| *Melissodes trinodis* | Soil | Su, Fa | 1,443 |
