# Supplementary material for: Ecosystem-Service Tradeoffs Associated with Switching from Annual to Perennial Energy Crops in Riparian Zones of the US Midwest
Source: PLoS One. 2013 Nov 6;8(11):e80093. doi: 10.1371/journal.pone.0080093 (PMC3819318; doi:10.1371/journal.pone.0080093)
Supplement: Table S3 — Average habitat quality scores (0 is worst and 1 is best) for nesting and foraging bees used in the InVEST Crop Pollination model. (DOC) [file pone.0080093.s006.doc]

| **Table S3. Average habitat quality scores (0 is worst and 1 is best) for nesting and foraging bees used in the InVEST Crop Pollination model.** | | | | | | | | |
| --- | --- | --- | --- | --- | --- | --- | --- | --- |
| Habitat | Nesting score | | | |  | Foraging score | | |
|  | Soil | Cavity | Hive | Wood |  | Spring | Summer | Fall |
| Continuous corn | 0.13 | 0 | 0 | 0.03 |  | 0.10 | 0.07 | 0.10 |
| Corn-soy rotation | 0.15 | 0 | 0 | 0.03 |  | 0.07 | 0.45 | 0.07 |
| Corn-alfalfa rotation | 0.37 | 0 | 0 | 0.03 |  | 0.12 | 0.35 | 0.17 |
| Continuous soybeans | 0.17 | 0 | 0 | 0.03 |  | 0.03 | 0.83 | 0.03 |
| Other annual crops | 0.17 | 0 | 0 | 0.03 |  | 0.03 | 0.83 | 0.03 |
| Small grains | 0.13 | 0 | 0 | 0.03 |  | 0.10 | 0.07 | 0.10 |
| Continuous alfalfa | 0.60 | 0 | 0 | 0.03 |  | 0.13 | 0.63 | 0.23 |
| Orchards | 0.80 | 0.47 | 0.5 | 0.33 |  | 0.93 | 0.20 | 0.13 |
| Open water | 0 | 0 | 0 | 0 |  | 0 | 0 | 0 |
| Suburbs | 0.83 | 0.73 | 0.83 | 0.70 |  | 0.60 | 0.63 | 0.63 |
| City | 0.43 | 0.37 | 0.43 | 0.37 |  | 0.34 | 0.35 | 0.35 |
| Barren | 0.03 | 0 | 0.03 | 0.03 |  | 0.07 | 0.07 | 0.07 |
| Deciduous forest | 0.73 | 0.90 | 0.90 | 0.57 |  | 0.83 | 0.43 | 0.30 |
| Conifer forest | 0.47 | 0.67 | 0.67 | 0.07 |  | 0.20 | 0.13 | 0.17 |
| Grassland | 1 | 0.03 | 0.03 | 1 |  | 0.63 | 0.90 | 0.93 |
| Wetland | 0.10 | 0.17 | 0.17 | 0.63 |  | 0.40 | 0.47 | 0.37 |
